# *Escherichia coli* under Ionic Silver Stress: An Integrative Approach to Explore Transcriptional, Physiological and Biochemical Responses

**DOI:** 10.1371/journal.pone.0145748

**Published:** 2015-12-22

**Authors:** Claire Saulou-Bérion, Ignacio Gonzalez, Brice Enjalbert, Jean-Nicolas Audinot, Isabelle Fourquaux, Frédéric Jamme, Muriel Cocaign-Bousquet, Muriel Mercier-Bonin, Laurence Girbal

**Affiliations:** 1 Université de Toulouse, INSA, UPS, INPT, LISBP, Toulouse, France; 2 INRA, UMR792 Ingénierie des Systèmes Biologiques et des Procédés, Toulouse, France; 3 CNRS, UMR5504, Toulouse, France; 4 Luxembourg Institute of Science and Technology (LIST), Material Research & Technology Department (MRT), Belvaux, Luxembourg; 5 Faculté de Médecine Rangueil, Centre de Microscopie Electronique Appliquée à la Biologie (CMEAB), Toulouse Cedex, France; 6 INRA, UAR1008, CEPIA, Nantes, France; 7 Synchrotron SOLEIL, Gif-sur-Yvette, France; National Cheng Kung University, TAIWAN

## Abstract

For a better understanding of the systemic effect of sub-lethal micromolar concentrations of ionic silver on *Escherichia coli*, we performed a multi-level characterization of cells under Ag^+^-mediated stress using an integrative biology approach combining physiological, biochemical and transcriptomic data. Physiological parameters, namely bacterial growth and survival after Ag^+^ exposure, were first quantified and related to the accumulation of intracellular silver, probed for the first time by nano secondary ion mass spectroscopy at sub-micrometer lateral resolution. Modifications in *E*. *coli* biochemical composition were evaluated under Ag^+^-mediated stress by *in situ* synchrotron Fourier-transform infrared microspectroscopy and a comprehensive transcriptome response was also determined. Using multivariate statistics, correlations between the physiological parameters, the extracellular concentration of AgNO_3_ and the intracellular silver content, gene expression profiles and micro-spectroscopic data were investigated. We identified Ag^+^-dependent regulation of gene expression required for growth (e.g. transporter genes, transcriptional regulators, ribosomal proteins), for ionic silver transport and detoxification (e.g. *copA*, *cueO*, *mgtA*, *nhaR*) and for coping with various types of stress (*dnaK*, *pspA*, *metA*,*R*, oxidoreductase genes). The silver-induced shortening of the acyl chain of fatty acids, mostly encountered in cell membrane, was highlighted by microspectroscopy and correlated with the down-regulated expression of genes involved in fatty acid transport (*fadL*) and synthesis/modification of lipid A (*lpxA* and *arnA*). The increase in the disordered secondary structure of proteins in the presence of Ag^+^ was assessed through the conformational shift shown for amides I and II, and further correlated with the up-regulated expression of peptidase (*hfq*) and chaperone (*dnaJ*), and regulation of transpeptidase expression (*ycfS* and *ycbB*). Interestingly, as these transpeptidases act on the structural integrity of the cell wall, regulation of their expression may explain the morphological damage reported under Ag^+^-mediated stress. This result clearly demonstrates that the cell membrane is a key target of ionic silver.

## Introduction

Ionic silver (Ag^+^) has been reported to exhibit strong biocidal activity in a wide range of bacteria and fungi, even at low micromolar concentrations [1–6]. Since ancient times, silver and its derived compounds have been empirically exploited for their therapeutic effect on clinical diseases (burn wounds, skin infections, newborn eye gonorrhea [7, 8]). In recent decades, silver derivatives have been used for new medical applications (e.g., silver sulfadiazine cream, silver-coated nylon fibers, dressings or catheters), notably against antibiotic-resistant bacteria [4].

The precise mechanisms of ionic silver antibacterial action are not fully understood. Several modes of action have been proposed, the most likely being the interaction of the Ag^+^ ions with electron-donating groups such as thiols, carboxylates, amides, imidazoles, indoles and hydroxyls [1, 9–11]. This type of chemical interaction could target membrane-bound and cytoplasmic proteins and enzymes, as well as bases in DNA. These interactions have been reported to cause (i) structural changes in the bacterial membranes [1], (ii) inhibition of the enzymes of the respiratory chain, and consequently, decoupling of respiration from ATP synthesis [12], (iii) formation of reactive oxygen species [13, 14], and (iv) lesions in DNA, affecting chromosome replication ability [1]. A recent study by our group was performed on the model bacterium *Escherichia coli* by synchrotron Fourier-transform infrared (sFTIR) microspectroscopy to elucidate the biochemical effects of ionic silver at single-cell scale [15]. These results showed that the observed biochemical changes were closely dependent on the concentration of Ag^+^ (lethal vs. sub-lethal concentration), growth conditions (stress applied from cell inoculation on, or as a pulse in the mid-exponential phase), and in some conditions, were associated with morphological damage. Indeed, the inhibitory action of silver ions was shown to be targeted against lipids and, at high concentrations, against proteins through a loss in α-helix conformation. The heterogeneity of Ag^+^-mediated effects within the bacterial population was also highlighted. In another study performed at the level of gene expression [16], the effect of silver ions on *E*. *coli* cells was compared to that of copper ions, highlighting shared up-regulation of genes coding for efflux pumps (i.e., up-regulation of the genes coding for CopA, a P-type ATPase efflux pump, and for CusCFBA, a Cu^+^ efflux pump) and a detoxifying oxidase (i.e., up-regulation of the gene coding for the CueO oxidase). More recently, the response of *E*. *coli* to nano-sized silver was investigated using the same type of transcriptomic approach [17]. This study provided evidence that exposure to this form of silver stimulates anaerobic respiration (i.e., up-regulation of anaerobic respiration-related reductases) and induces up-regulation of copper/silver resistance genes, including *cusBCF*, *copA* and *cueO*. However, a comprehensive understanding of the effect of ionic silver on *E*. *coli* bacteria is still lacking.

In this context, following our previously described results [15], the present work was devoted to multi-level characterization of *E*. *coli* exposed to Ag^+^-mediated stress in which an integrative biology approach, based on the combination of physiological, biochemical and transcriptomic data sets, was used for the first time. Bacterial growth and survival after Ag^+^ exposure were first quantified and related to the accumulation of intracellular silver, detected by secondary ion mass spectroscopy at high lateral resolution (NanoSIMS) [18–20]. The whole transcriptomic response of *E*. *coli* cells under ionic silver-mediated stress was then characterized. Based on the modifications in *E*. *coli* biochemical composition observed by *in situ* synchrotron Fourier-transform infrared microspectroscopy and depicted in detail in [15], clear correlations were established between (i) variations in the cellular physiological parameters, (ii) variations in the biochemical characteristics of cell fatty acids and proteins, and (iii) regulation of gene expression. This challenging approach is shown in the present study to be powerful for determining key genetic markers of the *E*. *coli* response to ionic silver. The expression of genes involved in general growth-related functions was globally down-regulated whereas that of genes related to stress responses (metal ion stress, general stress and oxidative stress) was up-regulated. Regulation of gene expression after Ag^+^ exposure was also correlated with modifications in *E*. *coli*, including cell morphology and biochemical composition, such as acyl chain length and protein secondary structure.

## Materials and Methods

### Bacterial strain and growth conditions

The laboratory strain *E*. *coli* K12 MG1655 (genotype: F- lambda- ilvG- rfb-50 rph-1 [21] was used in this work. As previously described in [15], a first inoculum was obtained by adding 1 mL of stock culture to 5 mL of Luria Bertani broth (Biokar Diagnotics, PRS Panreac) in a 15-mL half-opened BD Falcon^™^ tube, followed by 8 h of incubation at 37°C under continuous shaking at 120 rpm. This preculture was then used to inoculate 40 mL of M9 medium (Merck Prolabo) complemented with 2.5 g/L of anhydrous glucose (GPR Rectapur) with approximately 1.5x10^7^ CFU/mL (corresponding to an optical density at 595 nm [OD_595 nm_] of 0.02). This second preculture was incubated overnight under the same conditions (37°C, 120 rpm), then used to inoculate cultures to test the effect of ionic silver. The AgNO_3_ concentrations used in all the following experiments described here were 0, 5.0, 6.5 and 8.5 μM. These cultures were performed at 37°C and 120 rpm in 200 mL of M9 broth complemented with 2.5 g/L of anhydrous glucose, inoculated at 10^8^ CFU/mL (corresponding to an OD_595 nm_ of 0.12).

### Ionic silver treatment of *E*. *coli* cultures

Ionic silver stock solutions were prepared from AgNO_3_ powder (Fisher Scientific Bioblock) as previously described in [15] and stored in the dark. The final tested concentrations were obtained by diluting the corresponding AgNO_3_ stock solution directly into the M9 growth medium. A control experiment was simultaneously performed by adding deionised water to the M9 broth. In both cases, a stabilization period of 24 h at 37°C and 120 rpm was included before cell inoculation. Bacterial growth kinetics were then monitored by measuring optical density at 595 nm (OD_595 nm_). This made it possible to calculate the specific growth rate μ (h^-1^) throughout the course of the experiment, notably the rate reached at sampling time (i.e., after 3 h of culture; μ_t = 3h_) and that of the entire exponential phase of growth (μ_expo_). Experiments for each condition were performed in triplicate.

Samples were taken after 3 h of culture (corresponding to the mid-exponential phase for the growth without silver) for each condition. Cells were harvested by centrifugation with a Hettich Universal 320R centrifuge at 4,000 rpm for 5 min at 4°C, and washed twice with sterile saline solution (0.15 M NaCl). After incubation of cells for 24 h at 37°C, the cultivable cells were enumerated by plating on LB agar medium and expressed as colony-forming units per millilitre (CFU/mL). To account for variations in the number of cells recovered from each culture, the results were normalised by the total cell concentration and expressed in CFU/mL/ODU. Reported data correspond to the mean ± SD values of duplicates from three independent experiments.

### Biochemical composition of *E*. *coli* cells subjected to treatments with different concentrations of ionic silver

Modifications in the biochemical composition of *E*. *coli* cells grown in the presence of the different concentrations of ionic silver under study were evaluated at single-cell scale by *in situ* synchrotron FTIR micro-spectroscopy using the SMIS beamline at the SOLEIL Synchrotron (Gif-sur-Yvette, France), as previously described in detail in [15]. Briefly, the spatial resolution reached the micrometre order (4.1 μm), thanks to the use of a zinc-selenide attenuated total reflectance hemispherical internal reflection element, and optimized signal detection allowed by the use of the synchrotron source. Twenty spectra were acquired for each condition tested. The main informative regions observed were (i) the region 3,100–2,700 cm^-1^ representative of fatty acids mostly encountered in cell membranes; (ii) the region 1,800–1,480 cm^-1^ assigned to C = O stretching vibration, and amide I and amide II groups characteristic of the peptide bonds of cell proteins; and (iii) the region 1,450–1,150 cm^-1^ attributed to PO_2_^-^ groups mostly found in nucleic acids and, in some cases, to C-O stretching modes. To improve the detection of shifts in the characteristic absorption bands, the sFTIR spectra were pre-processed by calculating the second derivative (Savitsky—Golay, order 3, 9 points) on the whole infrared range (4,000–800 cm^−1^).

### Elemental composition mapmaking of *E*. *coli* cells subjected to ionic silver treatments

Elemental maps of silver were obtained by NanoSIMS50 analysis from semi-thin sections of resin-embedded bacterial cells, by adapting an experimental protocol previously used for yeast cells [22]. Washed cells sampled after 3 h of culture were fixed for 1 h at 4°C using a 2.5% glutaraldehyde solution in cacodylate buffer (0.1 M, pH = 7.4), then harvested by centrifugation at 1,000 rpm for 10 min and embedded in a 1.5% agar solution. A second fixation step was performed for 16 h at 4°C with the same solution, followed by three washings in a cacodylate buffer (0.2 M, pH = 7.4). The post-fixation step was performed in a solution of osmium tetroxide (2%) in cacodylate buffer (0.2 M, pH = 7.4) for 2 h at room temperature, followed by three washings as previously described. Cell dehydration was achieved through a series of incubations in graded ethanol solutions (10 min each in 50%, 70% and 80%, then 2×15 min each in 90% and 95% ethanol). Infiltration and embedding were performed by incubating cells in several changes of LRWhite resin/ethanol mixtures, in which the concentration of resin was gradually increased to 100% (resin/ethanol 1:3, 1:2, 2:3 and pure resin, each bath for 12 h at 4°C). Samples were left to polymerize for 24 h at 60°C and finally sectioned into 300-nm thick slices (Ultracut Reichert) and mounted on silicon plots (Siltronix, Archamps, France).

Nano-secondary ion mass spectrometry (Nano-SIMS) analyses were then performed with a NanoSIMS50 (Cameca, Gennevilliers, France). The Cs^+^ primary beam was accelerated at 8 keV and used for rasterizing a 10 x 10 μm² area on the polarized sample surface (– 8 keV). The primary ion intensity was 1.0–0.8 pA corresponding to a probe working diameter in the range of 50 to 80 nm (lateral resolution; [19]) and leading to the erosion of 1–2 nm of matter. The masses simultaneously recorded using a multi-detection system were ^12^C^14^N^−^ (m = 26.00307), ^31^P^−^ (m = 30.97376), ^32^S^−^ (m = 31.97207) and ^107^Ag^−^(m = 106.905095), whose intensities were expressed in counts per second (c/s). The distribution maps were recorded in a pixel format of 256 x 256 image points, with an acquisition time of 40 ms per pixel. Mass resolution (M/ΔM) was above 5,000 and mass calibration of silver was achieved using silver foil (Goodfellow, Huntingdon, UK). The isotopic ratio of ^107^Ag^-^ to ^109^Ag^-^ was checked to ensure that no isobaric interference had caused inaccurate measurements.

For each sample, at least 30 bacterial cells were selected on different images and analyzed individually. Data were treated with the module ImageJ plugin to process and analyze the captured images [23]. After selection of regions of interest (ROI), which here corresponded to a single bacterium, the total number of counts of each element detected by SIMS was calculated pixel by pixel. Digital masks of bacterial cells were defined using the ^12^C^14^N^−^ image, which provided both the highest contrast and widest ranges of grey values. Phosphorus and sulphur signals were indicative of cell components [22, 23]. The ^107^Ag^−^signal enabled the detection of silver atoms present in the cells. To obtain comparable Ag intensity values independently from variations in the intensity of the NanoSIMS50 signal occurring during measurements, the ratio of ^107^Ag^−^to ^12^C^14^N^−^ intensity counts (Ag/CN, dimensionless) was calculated for each selected bacterium, based on the assumption of a relatively constant ^12^C^14^N^−^ content in the selected area (i.e., the surface of the cell section) [18]. Although the real Ag concentration within cells cannot be assessed, this method made it possible to compare silver uptake between the different samples under study. The mean ± SD of these measurements was then determined, resulting in a “mean Ag/CN intensity ratio” for each sample.

### Microarray procedure and gene expression data analysis

At 3 h of culture, 60 mL of culture (at OD_595 nm_ = 0.73 ± 0.07, 0.63 ± 0.02, 0.12 ± 0.01 and 0.11 ± 0.02 for cultures subjected to 0, 5.0, 6.5 and 8.5 μM AgNO_3_, respectively) were sampled, placed in 50-mL BD Falcon^™^ tubes and centrifuged at 4,000 rpm for 5 min at 4°C, after which the pellet was frozen in liquid nitrogen. The pellet was resuspended in 1 mL of TE buffer (Tris-HCl 10 mM, pH 8, EDTA 1 mM, 1 mg lysozyme) and incubated for 5 min at room temperature. Total RNA was extracted with an RNeasy midi kit (Qiagen), including the DNase I treatment described in the manufacturer’s instructions. The total quantity and integrity of RNA were checked with a Nanodrop^®^ and an Agilent BioAnalyzer, respectively. A double strand cDNA synthesis kit (InvitroGen) was used to produce cDNA from 1.5 μg of total RNA. Following the manufacturer’s instructions, 1 μg of cDNA was labeled using the one color DNA labeling kit and 2 μg of labeled cDNA were hybridized onto *E*. *coli* K-12 gene expression arrays (Nimblegen, Roche) for 17 h at 42°C. Arrays were later washed and scanned using a MS200 Microarray Scanner (Nimblegen, Roche). The pictures were analyzed with DEVA 1.2.1 software. All array procedures were performed by the GeT-Biopuces platform (http://get.genotoul.fr). Raw and processed data were deposited in the Gene Expression Omnibus data repository (GSE67735).

Raw probe intensities (three replicates for each concentration of ionic silver) were analyzed in an R computing environment [24] using the affy [25] and limma [26] package of BioConductor. Raw data were subjected to an RMA-base background correction [27]. After background correction, intra-replicate quantile normalization was performed for each concentration of ionic silver. A set of probes in whose background the ranks were roughly the same across all the 12 arrays was selected. The median value of the intensities of the probe set for each array was used as a scaling factor for normalisation between growth conditions. After normalisation, the expression of a gene was calculated by RMA summarisation [27] for each concentration of ionic silver. Differential gene expression was evaluated using a modified t-test combined with an empirical Bayes method [28]. The p-values for the contrast of interest were adjusted for multiple testing by the *Benjamini—Hochberg* False Discovery Rate (FDR) [29]. A p-value threshold of 1% and a fold change < 0.5 or > 2 were used for differential expression. Agglomerative hierarchical clustering of the biological samples on the extracted genes for heat map display was derived using the Euclidean distance as the similarity measure and the Ward agglomeration method using the stats R package [24]. Gene ontology enrichment analysis of the extracted genes was performed using the topGO R package [30].

### Data integration

To identify and interpret correlation patterns in several large data matrices from the same biological system under investigation, sparse partial least squares regression (sPLSR) [31] was applied using the mixOmics R package [32]. To this end, three data sets were used:

1. Variations in internal and external silver contents and physiological parameters from the designed study (in replicates) corresponding to Table 1.
2. The gene expression data of size (12x3486) containing expression values (in triplicate for each concentration of ionic silver) for the 3,486 genes differentially expressed in at least one of the three concentration of ionic silver under ionic-silver mediated stress. Two gene expression subsets were also used, restricted to genes either involved in fatty acid metabolism (size 12x106) or protein folding (size 12x104).
3. The sFTIR data of size (75x271) were provided by the second derivative spectra from the sFTIR microscopy data, as indicated above and detailed in [15]. The three wavenumber regions of interest were selected for integration: (i) 3,100–2,700 cm^-1^ (fatty acids), (ii) 1,800–1,480 cm^-1^ (representative characteristic of C = O stretching vibration, amide I and amide II bands of peptide bonds) and (iii) 1,450–1,150 cm^-1^ (PO_2_^-^ groups and, for a part, C-O stretching modes).

**Table 1:** Physiological parameters of *E*. *coli* cultures (in M9 medium supplemented with 2.5 g/L of glucose) as a function of the concentration of extracellular AgNO_3_. The value μ_t = 3h_ corresponds to the specific growth rate calculated between 2.5 and 3.5 h of culture. The μ_expo_ value is the specific growth rate over the entire exponential growth phase (start and end specified in brackets) after the beginning of growth. The mean ± SD values of three independent experiments are given, except at a concentration of 6.5 μM AgNO_3_ for which the values of all three experiments are detailed.

[AgNO_3_] in the medium (μM)	Lag time (h)	μ_t = 3h_ (h^-1^)	Number of cultivable cells at 3 h (CFU/mL/ODU)	μ_expo_ (h^-1^)	Maximum absorbance (at 595 nm)
0 (control)	0.5 ± 0.0	0.62 ± 0.01 (from 2.5 to 3.5 h)	5.2 ± 1.3 x 10^8^	0.63 ± 0.01 (from 0.5 to 5.5 h)	3.31 ± 0.12
5.0	0.5 ± 0.0	0.63 ± 0.03 (from 2.5 to 3.5 h)	4.3 ± 1.2 x 10^8^	0.59 ± 0.03 (from 0.5 to 5.5 h)	3.19 ± 0.11
6.5	9	0	2.4 x 10^8^	0.54 (from 10.0 to 14.5 h)	3.13
6.5	17	0	2.3 x 10^8^	0.44 (from 18.0 to 24.5 h)	3.36
6.5	> 24	0	4.3 x 10^7^	0	0.11
8.5	> 24	0.0 ± 0.0	1.1 ± 0.2 x 10^7^	0.0 ± 0.0	0.10 ± 0.01
100^a^	—	0.0 ± 0.0	0.0 ± 0.0	0.0 ± 0.0	—

^*a*^: values from [15].

In sPLSR, the two-block data matrices to be integrated were denoted X(n x p) and Y(n x q), where p and q are the total number of variables measured on the same n subjects. When two data sets to be integrated displayed different sample numbers n, the dataset with the lowest sample number was completed by a bootstrap method to reach the higher n value. We analysed the data sets using sPLSR in an attempt to explain/predict the Y variables with respect to X and to perform simultaneous variable selection in the two datasets [31]. The optimum number of PLS components in the sPLSR models and the number of variables to be selected on each dimension were found by cross-validation by calculating the Q2 criterion and the minimum root mean square error (RMSE), respectively. Correlation circle plots and relevance networks resulting from the sPLSR approach [33] were used to help interpret the results. To highlight the strongest variable associations only, variables with a correlation greater than 0.5 in absolute value were chosen to infer the relevance networks (see figure legends). This threshold was arbitrarily chosen to obtain biologically interpretable networks that were neither too sparse nor too dense. The networks obtained were then used as inputs for Cytoscape [34] for visualisation.

To correlate the intracellular silver content and physiological parameters with biochemical changes within bacterial cells, sPLSR analysis was performed with variations in AgNO_3_ concentrations and physiological parameters data as X and the selected sFTIR bands as Y. When sPLSR was used to integrate the gene expression data and the sFTIR spectra, the sPLSR was run with the gene expression as X and the sFTIR spectra as Y. Variations in AgNO_3_ concentrations and physiological parameters and gene expression were integrated by running sPLSR using the AgNO_3_ concentrations and physiological parameters data as X and the gene expression as Y.

## Results

### Physiological and biochemical responses of *E*. *coli* to ionic silver

Physiological parameters of *E*. *coli* cells grown in the presence of different concentrations of ionic silver ([AgNO_3_] = 5.0, 6.5 or 8.5 μM in the growth medium from inoculation) were assessed and compared to those of the control experiment (i.e., bacteria grown without any added silver) (Table 1). A concentration of 5.0 μM of AgNO_3_ had a weak effect on *E*. *coli* growth, as the cells reached a μ_expo_ of 0.59 ± 0.03 h^-1^ (0.63 ± 0.01 h^-1^ for the control). At a concentration of 6.5 μM of AgNO_3_, the three replicates exhibited quite different physiological behaviours. In one replicate, no growth was observed before the end of the experiment (i.e. 24 h), although bacterial cells were still able to grow on LB agar medium after washing (Table 1). In the two other replicates, exponential growth occurred at a lower μ_expo_ than the control (0.54 and 0.44 h^-1^) after a lag phase of 9 h and 17 h, respectively. At the end of these two cultures, the maximum concentration of biomass reached a similar value to that obtained at 5.0 and 0 μM. At a concentration of 8.5 μM of AgNO_3_, no bacterial growth was observed over a period of 24 h. Although these cells did not grow in the presence of ionic silver, after washing (i.e. removal of the silver), a significant number of cells was still cultivable (10^7^ CFU/mL/ODU). For the purpose of comparison, at a concentration of 100 μM of AgNO_3_, 100% of the cells were no longer cultivable, showing that 100 μM of AgNO_3_ was a lethal concentration (Table 1). Taken together, these results show that 6.5 μM is a pivotal value for the physiological effect of ionic silver, and that concentrations of up to 8.5 μM of AgNO_3_ can be considered as sub-lethal.

**Fig 1:**
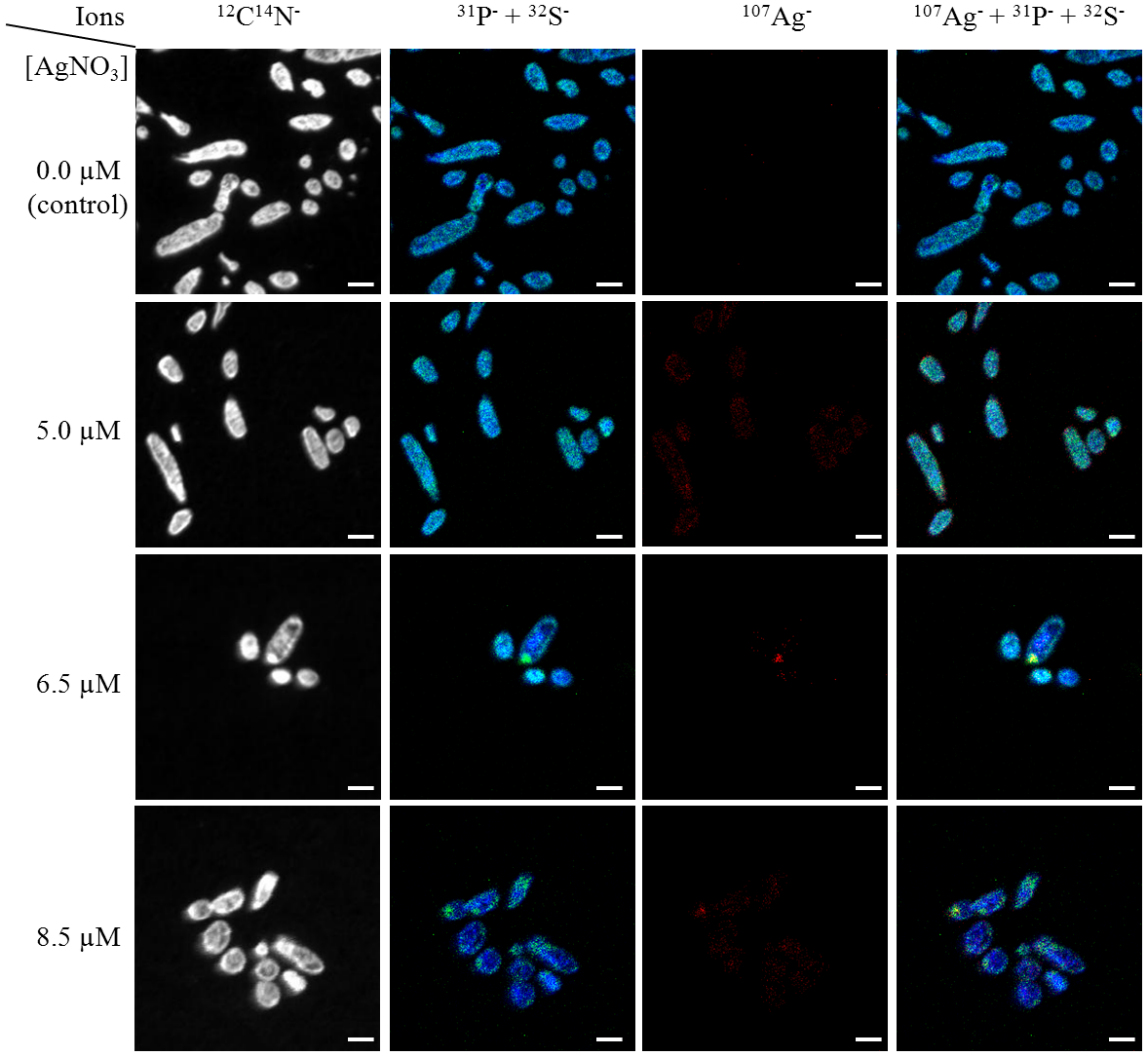
Distribution maps of CN (in grey), P (in blue), S (in green) and Ag (in red) elements detected by NanoSIMS analysis in *E*. *coli* cells after 3 h of culture without ionic silver (control) or in the presence of 5.0, 6.5 and 8.5 μM of AgNO_3_. The fourth column corresponds to overlapping of the three-coloured images (Composite Image mode [23]). Scale bar: 2 μm.

To visualise the presence of intracellular silver after 3 h of culture, the silver element was mapped by NanoSIMS on at least 30 individual cells in each sample (Fig 1). The distribution of the ^12^C^14^N^−^ cluster was simultaneously recorded as a “fingerprint” of bacterial cells, while the signals of phosphorus and sulphur made it possible to highlight interactions between the ionic silver and cell components (Fig 1). As shown in the images of bacterial cells grown in the presence of AgNO_3_, silver mainly overlapped sulphur. The distribution of phosphorus was quite homogeneous, which was consistent with its ubiquitous presence in the cell (nucleic acids, phosphorylated metabolites, etc.).

**Fig 2:**
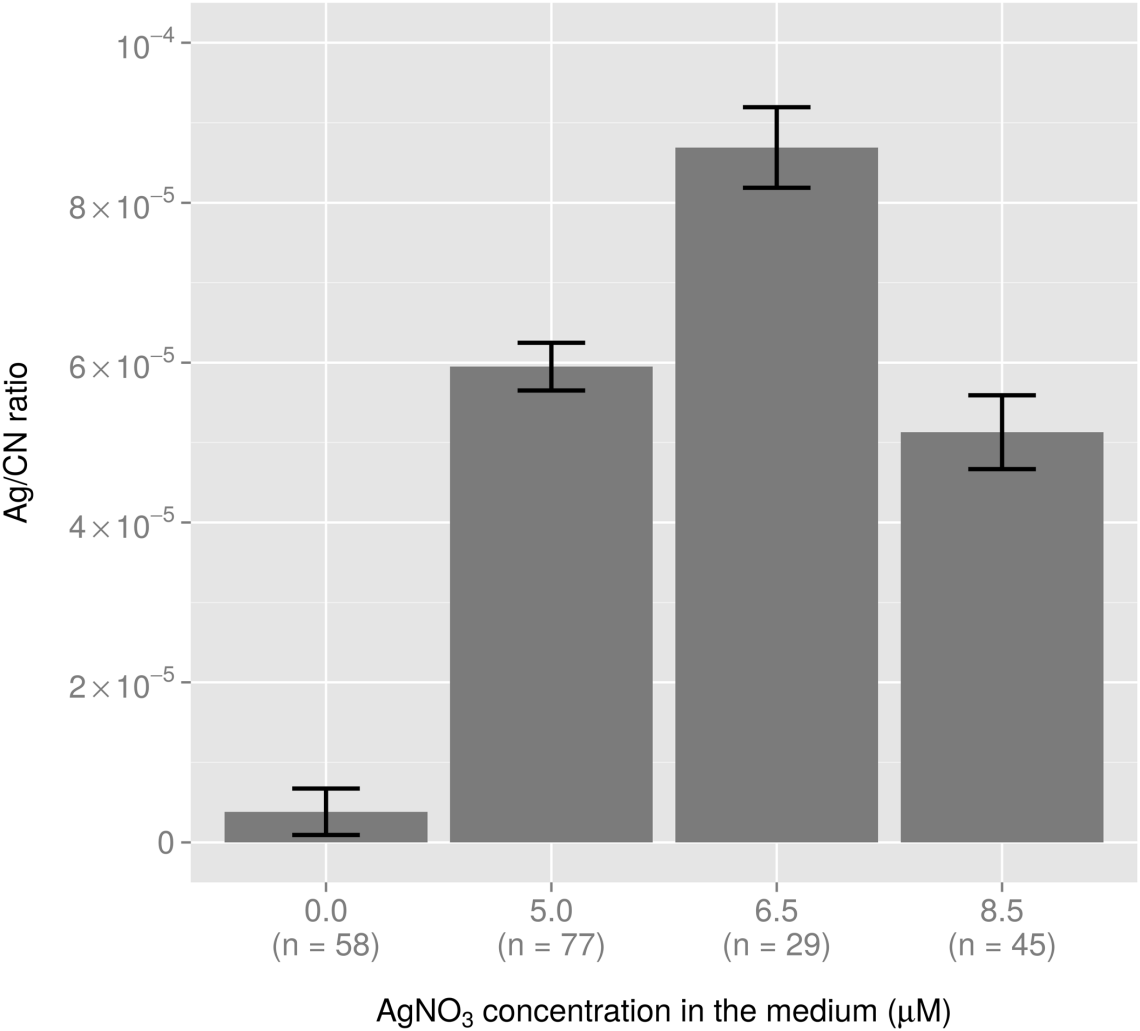
Variation in the Ag/CN ratio with the extracellular AgNO_3_ concentration deduced from NanoSIMS analysis. For each condition, the number of bacterial cells used for calculation (n) is indicated.

To obtain a semi-quantitative value, the intensity ratio between the two signals (Ag/CN) was then calculated for each cell [35] and averaged for each group (Fig 2). A significant increase in the intracellular silver content was observed in cells treated with 5.0 μM AgNO_3_, as the Ag/CN ratio was multiplied by a factor of 15 compared to untreated cells (Fig 2). The Ag/CN ratio measured in bacteria treated with 6.5 μM of AgNO_3_ increased by a factor 1.5 compared to the cells treated with 5.0 μM of AgNO_3_, while it was of the same order of magnitude as the latter in cells treated with 8.5-μM of AgNO_3_ (Fig 2).

### Integrative analysis of the physiological parameters and the biochemical response of *E*. *coli* after Ag^+^ exposure

The first approach used to integrate the data associated variations in the concentration of extracellular AgNO_3_, intracellular silver content (reflected by the Ag/CN ratio), physiological parameters (specific growth rate μ_t = 3h_ and the number of cultivable cells expressed in CFU/mL/ODU) and modifications in *E*. *coli* biochemical composition, evaluated by *in situ* sFTIR micro-spectroscopy at the single-cell scale (data and peak assignments from [15]; see the experimental section for further details). Raw sFTIR spectra recorded on bacteria grown in the absence or in the presence of ionic silver at sub-lethal concentrations, are presented in S1 Fig. The peaks were identified based on our previous study [15].

**Fig 3:**
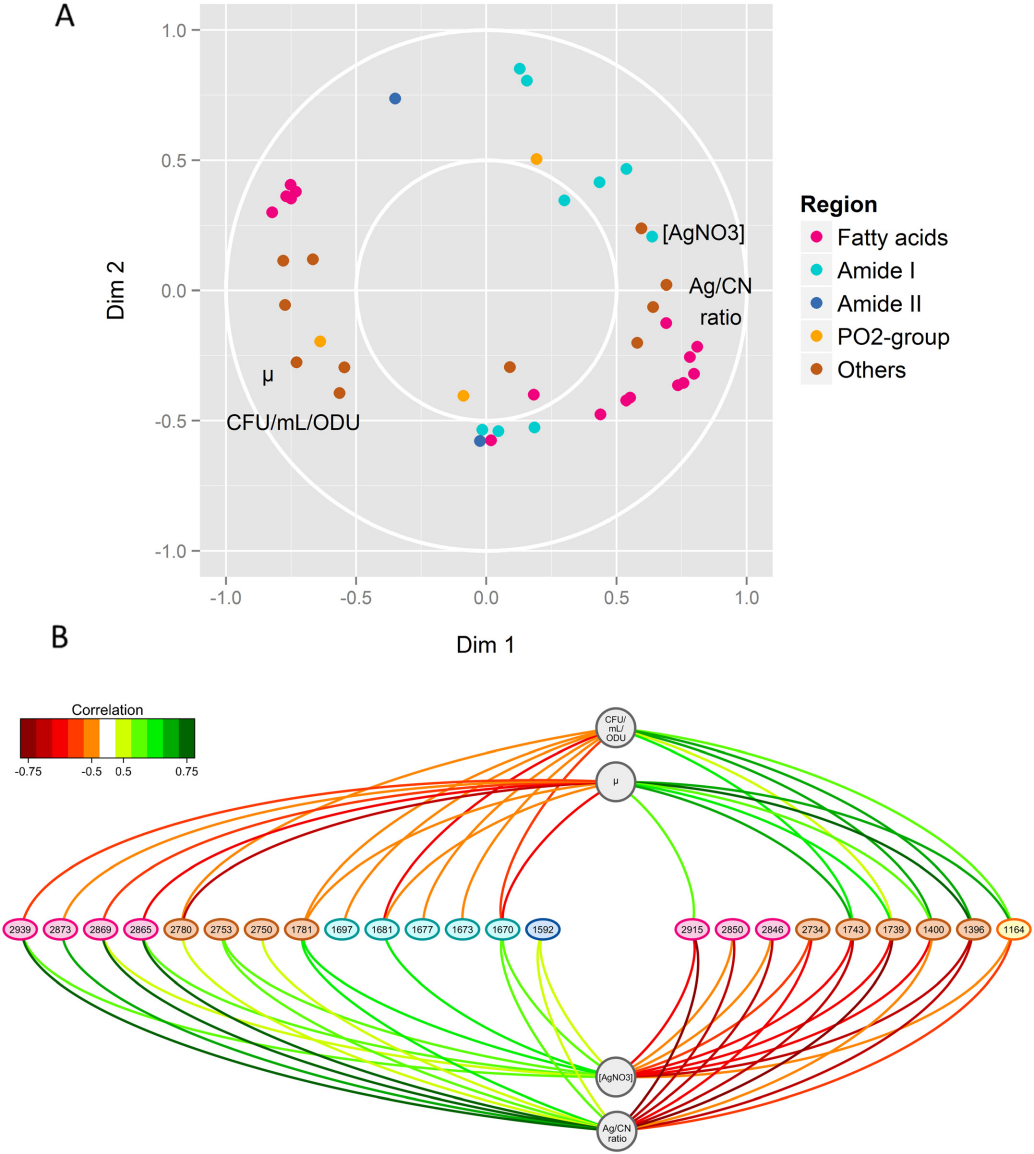
Results of the sPLSR integrative approach between the Y matrix, corresponding to AgNO_3_ concentrations and physiological parameters (i.e., specific growth rate μ_t = 3h_, number of cultivable cells (CFU/mL/ODU) and intracellular silver content illustrated by the Ag/CN ratio at 3 h culture) and the X matrix composed of the selected sFTIR data (i.e. the regions 3,100–2,800 cm^-1^, 1,700–1,600 cm^-1^, 1,600–1,480 cm^-1^ and 1,300–1,150 cm^-1^, representative of fatty acids, C = O stretching vibration, amide I and amide II bands and PO2- groups and C-O stretching mode, respectively). The regions 2,800–2,700 cm^-1^, 1,800–1,700 cm^-1^ and 1,480–1,300 cm^-1^ are grouped under “Others”. (A) Correlation circle plots for dimensions 1 and 2. The subsets of correlated wavenumbers are represented by thick points coloured according to the legend, while the physiological parameters are represented by their name in black. (B) Relevance networks resulting from the sPLSR approach. A threshold of 0.5 was used for relevant correlations. Green and red edges indicate positive and negative correlations, respectively. Physiological parameters and wavenumbers are represented as circles and ellipses, respectively.

As expected, the intracellular silver content was correlated with the concentration of extracellular AgNO_3_, and both were anti-correlated with the specific growth rate μ_t = 3h_ and with the number of cultivable cells (Fig 3A). Furthermore, the intracellular silver content was correlated with wavenumber characteristics of fatty acids and peptide bonds (Fig 3A and 3B). In particular, the Ag/CN ratio was positively correlated with the wavenumbers 2,873, 2,869 and 2,865 cm^-1^, assigned to the symmetric C-H stretching of—CH_3_ groups in lipids, but negatively correlated with the wavenumbers 2,850 and 2,846 cm^-1^, representative of the C-H symmetric stretch of >CH_2_ groups. This showed that the presence of silver in *E*. *coli* cells led to an increase in CH_3_ over CH_2_ groups, corresponding to a shortening of the aliphatic chains of bacterial lipids. Positive and negative correlations of the Ag/CN ratio were also observed with respectively, the up-shift (2,939 cm^-1^) and the downshift (2,915 cm^-1^) of the asymmetric C-H stretching of >CH_2_ groups (2,930 cm^-1^), suggesting an increased proportion of “cis” over “trans” conformation in unsaturated fatty acids of cells subjected to ionic silver-mediated stress.

In the spectral range from 1,800 to 1,700 cm^-1^, it will be noted that the wavenumbers 1,743 and 1,739 cm^-1^, which are characteristic of C = O stretching vibrations in lipid esters [36], were positively correlated with the specific growth rate and the number of cultivable cells but negatively correlated with the intracellular silver content and the extracellular concentration of AgNO_3_ (Fig 3B). Concerning the peptide bond, the intracellular silver content and the extracellular concentration of AgNO_3_ were correlated with wavenumber 1,670 cm^-1^, the AgNO_3_ concentration with 1,681 cm^-1^ while 1,697, 1,677 and 1,673 cm^-1^ were anti-correlated with the number of cultivable cells (Fig 3B). These wavenumbers, assigned to anti-parallel pleated β-sheets of amide I, unordered random coil and turn forms of amide I [36], suggest a disordered secondary structure of proteins after Ag^+^ exposure. Some nucleic acid damage is possible as wavenumber 1,592 cm^-1^ [36] was positively correlated with the Ag/CN ratio and with the extracellular AgNO_3_ concentration (Fig 3B). The extracellular concentration of AgNO_3_ and intracellular silver content were positively correlated with wavenumbers 2,750 and 2,753 cm^-1^ and negatively correlated with 2,734 cm^-1^, while the peak at 2,780 cm^-1^ was positively correlated with the Ag/CN ratio and negatively correlated with the specific growth rate and the number of cultivable cells (Fig 3B). These four wavenumbers are characteristic of N-H stretching [36]. The wavenumbers 1,400 and 1,396 cm^-1^, attributed to symmetric CH_3_ bending of the methyl groups of proteins, and the wavenumber 1,164 cm^-1^ attributed to C-O stretching in serine, threonine and tyrosine of proteins [36], were positively correlated with the specific growth rate and the number of cultivable cells but negatively correlated with the Ag/CN ratio and the extracellular concentration of AgNO_3_ (Fig 3B). Finally, wavenumber 1,781 cm^-1^ could not be attributed to any biochemical component of the cell.

### Transcriptional response of *E*. *coli* to ionic silver

To analyse the impact of ionic silver on global gene expression, transcriptomic analyses were performed after a 3-h exposure to concentrations of 5.0, 6.5 and 8.5 μM of ionic silver, and compared to control conditions, i.e. without the addition of any silver. At a concentration of 5.0 μM of silver, the yield of total extracted RNA was not affected whereas at concentrations of 6.5 and 8.5 μM, 2-fold less yield was measured. In addition, at these two AgNO_3_ concentrations, the proportion of mRNA to total RNA (estimated by summing all the spot intensities in the array) was reduced more than 3-fold. The reduced proportion of mRNA may be linked to a decrease in transcription and/or an increase in mRNA degradation at the highest concentrations of ionic silver.

Differentially expressed genes were determined by comparing expression under the three above mentioned conditions. A total of 3,486 genes (corresponding to 81% of the total of 4,254 *E*. *coli* genes) were significantly differentially expressed (p-value < 1% with a fold change < 0.5 or > 2) at least in one of the three comparisons (S1 Table). A comparative transcriptomic analysis was first performed to determine the extent of overlap between our transcriptomic response to ionic silver (this study) and transcriptomes of *E*. *coli* cells responding to other stressful conditions depicted in the literature (i.e., subjected to from sub-lethal to lethal concentrations of other metal ions, under oxidative conditions or after a heat shock) (Table 2). High correlations were observed between 6.5 μM AgNO_3_, 8.5 μM AgNO_3_, 6 mM H_2_O_2_ and heat shock at 45°C (correlation coefficients > 0.35). Under all these conditions, the imposed stress led to growth arrest. No strong similarity was observed between our silver ion response and the other metal ion responses reported in the literature except between 8.5 μM AgNO_3_ and 2 mM copper ion (correlation coefficient of 0.15) (Table 2). It is interesting to note that the 5.0 μM AgNO_3_ treatment did not correlate with the 8.5 μM treatment and only slightly with the 6.5 μM treatment. These results suggest that the transcriptomic response really only took place at a concentration of more than 5 μM.

**Table 2:** Pearson correlation between transcriptomic matrix for a selection of stress and ion response conditions. The similarity between the log2 matrix of the gene expression ratio vs. the untreated control was determined under the following conditions: oxidative stress (6 mM H_2_O_2_, time 10 minutes from [53]), heat shock (45°C, time 10 minutes from [54]), silver ion response (this study, in bold), copper ion response (2 mM, from [55]), cadmium ion response (0.1 mM, from [56]), zinc ion response (0.2 mM, from [48]) and cobalt ion response (0.25 mM, from [57]). Correlations coefficients ≥ 0.35 are in bold.

	H_2_O_2_, 6 mM	HS, 45°C	Ag, 8.5 μM	Ag, 6.5 μM	Cu, 2 mM	Cd, 0.1 mM	Zn, 0.2 mM	Ag, 5 μM	Co, .25 mM
H_2_O_2_, 6 mM	**1.00**	**0.54**	**0.47**	**0.35**	0.19	0.04	-0.01	-0.16	-0.05
HS, 45°C	**0.54**	**1.00**	**0.52**	**0.45**	0.24	0.10	-0.05	-0.14	-0.02
Ag, 8.5 μM	**0.47**	**0.52**	**1.00**	**0.78**	0.15	0.09	0.07	0.02	-0.02
Ag, 6.5 μM	**0.35**	**0.45**	**0.78**	**1.00**	0.25	0.23	0.09	0.14	0.04
Cu, 2 mM	0.19	0.24	0.15	0.25	**1.00**	0.11	0.13	-0.05	0.07
Cd, 0.1 mM	0.04	0.10	0.09	0.23	0.11	**1.00**	-0.05	0.06	0.05
Zn, 0.2 mM	-0.01	-0.05	0.07	0.09	0.13	-0.05	**1.00**	0.09	0.02
Ag, 5 μM	-0.16	-0.14	0.02	0.14	-0.05	0.06	0.09	**1.00**	0.06
Co, 0.25 mM	-0.05	-0.02	-0.02	0.04	0.07	0.05	0.02	0.06	**1.00**

**Fig 4:**
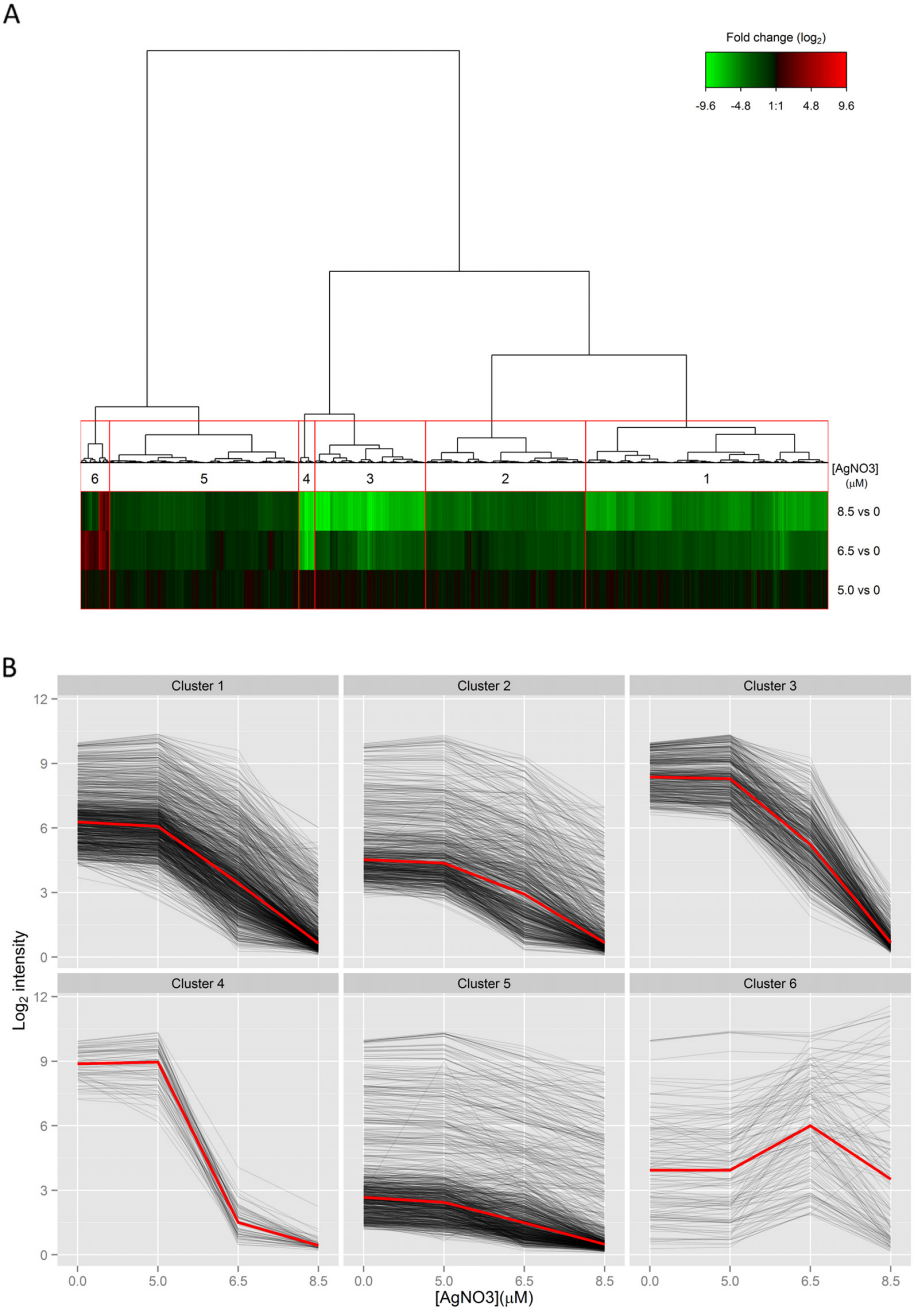
Ionic silver-mediated expression regulation. (A) Hierarchical clustering of expression ratios between growth conditions with 5.0, 6.5 or 8.5 μM of AgNO_3_ in the medium and the control (i.e., no ionic silver added) for the 3,486 differentially-expressed genes in one of the three ionic-silver mediated stress conditions. The resulting heat map shows the samples in rows and the genes in columns, red indicates up-regulation, green down-regulation, and black no change. (B) The expression of genes in each cluster is plotted as a function of the concentration of AgNO_3_ in the medium to show the general trend of expression regulation. The red line in the individual clusters represents the median calculated from the expression levels of the genes. Cluster 1: 1,131 genes, cluster 2: 746 genes, cluster 3: 516 genes, cluster 4: 76 genes, cluster 5: 881 genes and cluster 6: 136 genes.

To get further insight into the progressive regulation of gene expression with an increase in the concentration of AgNO_3_, a clustering procedure was implemented to distinguish different gene expression regulation profiles (Fig 4A). Clustering of gene expression ratios between conditions (5.0 vs. 0, 6.5 vs. 0 and 8.5 vs. 0 μM AgNO_3_) differentiated six clusters (Fig 4B, S1 Table). All clusters exhibited a weak transcriptomic response in the first comparison “5.0 vs. 0 μM AgNO_3_” confirming the lack of strong transcriptomic response at 5.0 μM. Above 5.0 μM, the profiles differed in the six clusters. The first five clusters (1 to 5) generally corresponded to down-regulated gene expression over the whole range of AgNO_3_ concentrations but differed in the magnitude of the decrease between 8.5 μM AgNO_3_ and 0 μM: clusters 1 and 2 corresponded to moderate decreases (average 15-fold, 53% of differentially expressed genes), cluster 5 included small decreases (average 4-fold, 25% of differentially expressed genes) while the very large decreases (average 200-fold) corresponding to 17% of the differentially expressed genes were grouped in clusters 3 and 4. Cluster 6 (136 genes, 4% of differentially expressed genes) contained genes up-regulated at 6.5 μM and/or 8.5 μM of AgNO_3_. Therefore, except for 4% of the differentially expressed genes, gene expression was down-regulated in the presence of ionic silver at a concentration > 5.0 μM. This is in agreement with the smaller proportion of mRNA of total RNA at high AgNO_3_ concentrations.

We then examined the function of genes whose regulation differed from the general trend of moderate down-regulation. Enrichment in functional category was thus determined (p-value < 0.05) in clusters 3 and 4 of strongly down-regulated expression, in cluster 5 of less than the average down-regulated expression, and in cluster 6 of up-regulated gene expression (Table 3). Pathways related to growth, i.e. metabolic processes, transports, membrane biogenesis, cell shape regulation and the translational process, were over-represented in the strongly down-regulated subgroup of genes.

**Table 3:** Functional analysis of the genetic response of *E*. *coli* cells to ionic silver. Selected functional categories enriched (p-value < 0.05) in groups of genes with generally strongly down-regulated expression (clusters 3 and 4), weakly down-regulated expression (cluster 5) and up-regulated expression (cluster 6) are included. For each category, associated genes are also listed.

Functional category	Gene
**Strong down-regulation (clusters 3 and 4)**	
**Metabolic process**	
Glycolysis	*sucA*, *lpd*, *eno*, *fbaAB*, *gpmAM*, *pykF*, *pgi*
Glyoxylate cycle	*aceA*,*B*,*K*, *iclR*
Galactitol	*gatA*,*B*,*C*,*D*
Amino acids	*folD*, *ilvH*,*I*,*D*,*B*, *hisA*,*B*,*C*,*D*,*F*,*G*,*H*,*I*, *trpC*,*D*,*E*, *aroA*,*C*,*G*,*K*, *pheA*
Bases	*adk*, *fold*, *guaA*, *gmk*, *purB*,*C*,*D*,*F*,*K*,*H*,*L*,*M*,*N*,*T*,*U*, *carA*,*B*, *pyrCDGH*, *relA*, *spoT*, *thyA*
Glycogen energy reserve	*glgB*,*C*,*P*,*X*
**Transport**	
Maltose	*malE*,*F*,*K*
Proteins and peptides	*dppA*,*B*,*C*,*D*,*F*, *oppA*,*B*,*C*,*D*,*F*, *secA*,*B*, *lolB*,*C*,*E*, *tatA*,*B*
Iron	*fecIR*, *fep*, *fes*, *fhuC*,*E*, *feoAB*, *tolC*, *tonB*, *entACDE*, *fiu*, *efeO*, *fre*
Lipopolysaccharides	*lptA*,*B*,*C*,*D*
Electrons	*frdA*, *napA*, *narG*, *nirB*, *nuoA*,*B*,*C*,*E*,*F*,*G*,*H*,*I*,*J*,*K*,*L*,*M*,*N*, *cyoA*,*B*,*C*, *sucB*,*C*,*D*, *sdhA*,*C*,*D*, *aceA*,*B*,*K*, *lpd*, *dld*, *ppc*
Protons	*atpB*,*D*,*F*,*H*,*I*
**Membrane biogenesis**	
Lipopolysaccharides	*rfbAC*,*D*,*X*, *waaF*,*G*,*Q*, *wbbI*,*J*,*K*, *rffEG*, *rfaD*, *rmlB*, *rfc*, *rfe*, *glf*, *cld*, *lpxD*,*H*,*P*, *kdsD*, *glmU*, *gmhB*, *yijP*
Cell outer membrane	*bamA*,*D*, *lptD*, *hlpA*, *surA*
**Cell shape regulation**	*murB*,*C*,*E*, *mreB*,*C*,*D*, *rodZ*, *dacA*, *ftsW*, *mpl*, *mraY*, *yafK*, *glmU*, *ispU*
**Translation**	
Ribosome generation	*rsuA*, *rluB*,*D*,*E*
tRNA synthetase	*aspS*, *gltX*, *glyS*,*Q*, *hisS*, *lysS*, *metG*, *pheS*, *trpS*
Translation termination	*frr*, *prfA*,*B*,*C*
**Cell motility, chemotaxis and flagellum organization**	*cheA*,*B*,*R*,*W*,*Y*,*Z*, *dppA*, *flgA*,*B*,*C*,*D*,*E*,*F*,*G*,*H*,*I*,*J*,*K*,*L*,*M*,*N*, *flhA*,*C*, *fliC*,*D*,*F*,*G*,*H*,*I*,*J*,*K*,*L*,*M*,*N*,*O*,*S*,*T*, *frdA*, *motA*,*B*, *rbsB*, *tap*, *tar*, *tsr*, *yhjH*
**Small down-regulation (cluster 5)**	
**Transport**	
Copper ion transmembrane	*zupT*, *cusA*,*B*,*C*,*F*
Organic anion	*abgT*, *glcA*, *phnC*,*J*,*L*,*O*,*P*, *gltS*, *ychM*
Protein secretion (type II system)	*hofC*, *yghF*, *gspC*,*E*,*F*,*G*,*J*
Gluconate	*ygbN*, *dsdX*, *gntP*, *idnT*, *yjhF*
**Metabolic process**	
Aromatic compound	*paaF*,*G*,*H*,*J*, *hcaC*,*D*,*F*, *mhpC*,*D*,*E*,*F*, *tyrR*, *feaB*, *hcaB*, *tnaA*
Organic acids	*dgoA*,*D*,*K*, *yjjM*, *cynT*,*S*,*X*
Arabinose	*araA*,*C*, *sgbE*, *fucI*,*K*
Purines	*yagR*,*S*, *allA*,*C*, *xdhA*,*B*,*C*,*D*
Arginine	*speA*, *astB*,*C*,*D*,*E*
**Up-regulation (cluster 6)**	
**Response to stimulus**	
Stress	*clpB*, *dnaJ*,*K*, *htpG*,*X*, *lon*, *pspA*,*B*,*C*,*D*,*G*, *raiA*, *psiE*, *dps*, *ibpA*,*B*, *ydeI*, *rmf*, *bhsA*, *ariR*, *yodD*, *zinT*, *ygaW*, *ygiW*, *yhbO*, *ytfE*, *tisB*, *mntH*
Heat	*dnaJ*,*K*, *htpG*, *lon*, *pspA*, *ibpA*,*B*, *yhbO*
Hydrogen peroxide	*ydeI*, *ariR*, *yodD*, *ygiW*
Copper ion	*cueO*, *copA*
Cadmium ion	*zintT*, *ygiW*
Acidity	*asr*, *yodD*
**Transport**	
Divalent metal ion	*chaA*, *mgtA*, *mntH*
Inorganic anion	*yfeO*, *narU*, *sbp*
**Biofilm formation**	*ydeH*,*I*, *bhsA*, *ariR*, *yodD*, *msqR*, *ygiW*
**Homeostasis**	
Cellular sodium ion	*chaA*, *nhaA*
**Biosynthetic process**	
Methionine	*metA*,*B*,*F*,*R*, *mmuM*
Arginine	*argA*,*B*,*C*

In addition to the general down-regulation of growth-associated functions, specific responses to ionic-silver mediated stress were also observed. Cell motility, chemotaxis and flagellum organization were strongly affected. The most striking feature was the response to metal ions: genes involved in response to copper (*copA*, a copper efflux transport and *cueO*, a copper oxidase) and cadmium (*zinT*, a zinc and cadmium binding protein and *ygiW*, unknown function) were enriched in the group of up-regulated genes, whereas the Cu(I) efflux pump (*cusCFBA)* and the zinc transporter (*zupT*) were less down-regulated than average. We found up-regulated expression of transport of magnesium (*mgtA*), manganese (*mntH*) and calcium (*chaA* and *nhaA*). A general response to stress was observed with the enrichment of categories associated with stress in general and in particular heat shock proteins in the group of up-regulated genes. At the metabolic level, higher expression of the methionine- and arginine-related pathways was observed. Other pathways were also down-regulated by ionic silver, i.e. the catabolism of aromatic compounds, organic acids and sugars and the metabolism of purine. Altogether, these results revealed a marked impact of ionic silver on the gene expression profile, reducing general growth-related functions and favouring stress responses.

### Integrative analysis of the physiological parameters and the transcriptional response of *E*. *coli* after Ag^+^ exposure

**Fig 5:**
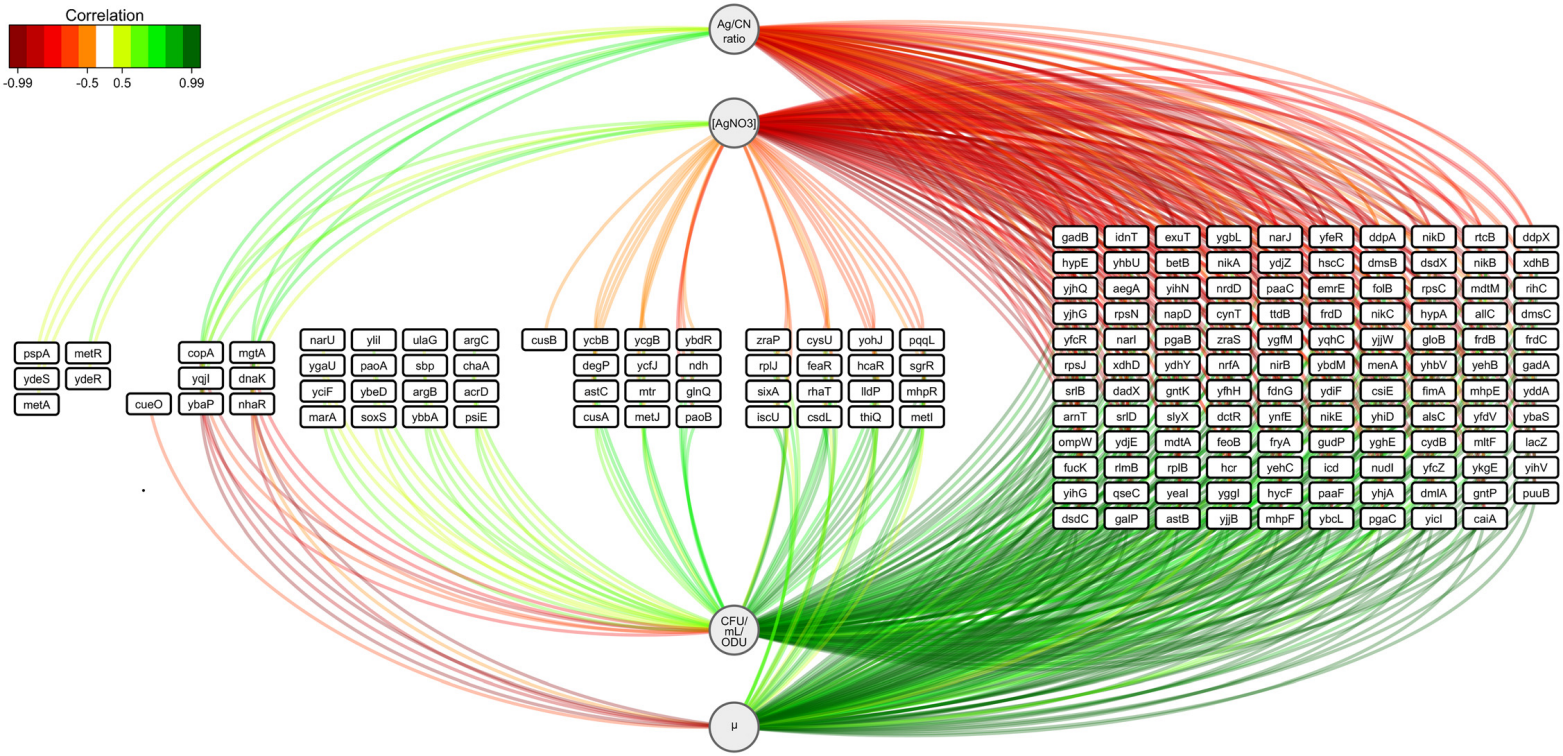
Relevance networks resulting from the sPLSR approach between the Y matrix corresponding to AgNO_3_ concentrations and physiological parameters (i.e., specific growth rate μ_t = 3h_, number of cultivable cells (CFU/mL/ODU) and intracellular silver content illustrated by the Ag/CN ratio at 3 h culture) and the X matrix representing the expressions of the 3,486 differentially expressed genes in one of the three AgNO_3_-mediated stress growth conditions. A threshold of 0.5 was used for relevant correlations. Green and red edges indicate positive and negative correlations, respectively. Physiological parameters are represented as circles and genes as rectangles.

To investigate the physiological significance of the Ag^+^-mediated modifications in gene expression, we analysed correlation patterns between the gene expression profiles, the extracellular silver concentration in the medium (AgNO_3_ concentration) and in the bacterial cells (Ag/CN ratio), and the physiological parameters (specific growth rate μ_t = 3h_ and the number of cultivable cells after 3 h of culture), using sparse partial least squares regression (sPLSR) analysis [31] (Fig 5). Among the correlated variables, most gene expression regulations were positively correlated with the specific growth rate and the number of cultivable cells and, as expected, negatively correlated with the external and intracellular silver contents. Expression was mostly down-regulated, and concerned (i) many transporters [32 genes including transport of copper (*cusAB*), nickel (*nikB*,*C*,*D*,*E*)], (ii) 10 transcriptional regulators (*dctR*, *dsdC*, *feaR*, *hcaR*, *metJ*, *mhpR*, *sgrR*, *yfeR*, *yfhH* and *yqhC*), (iii) genes involved in oxidation-reduction processes [27 genes including nitrite/nitrate reductase (*napD*, *narI*,*J*, *nirB* and *nrfA*), fumarate reductase (*frdB*,*C*,*D*), dimethyl sulfoxide reductase (*dmsB*,*C*), xanthine oxidoreductase (*xdhB*,*D*), hydrogenase (*hypA*,*E*) and cupric reductase (*ndh*)], (iv) genes coding for membrane/periplasmic proteins (*nikA*, *ompW*, *yihG*,*H*, *yjjB*, *yohJ* and *zraP*) and ribosomal proteins (*rplB*,*J* and *rpsC*,*J*,*N*). The AgNO_3_ concentration and the Ag/CN ratio were rarely positively correlated with the regulation of gene expression. All the regulations positively correlated with the AgNO_3_ concentration and the Ag/CN ratio were up-regulated in the presence of ionic silver. The genes were shown to be involved in responses to general stress *(dnaK* and *pspA*) and copper (*copA* and *cueO*), ion transport (magnesium *mgtA*, sodium *nhaR*), and methionine biosynthesis (*metA*,*R*) and putatively in iron homeostasis (*yqjI*) and in adhesion (*ydeS*,*R*). The combination of the different regulation of gene expression constitutes the genetic pattern of the physiological response of *E*. *coli* to Ag^+^-mediated stress.

### Integrative analysis of the biochemical and transcriptional responses of *E*. *coli* after Ag^+^ exposure

As shown above, ionic silver stress mainly affected fatty acids (chain length, saturation and conformation) and peptide bonds (secondary structure). We next focused on regulation of the expression of genes specifically involved in fatty acid metabolism and protein folding in the presence of Ag^+^ ions to provide additional insights into Ag^+^-related functional genomics.

Regarding fatty acid metabolism, we observed down-regulation of genes involved not only in the initiation of fatty acid biosynthesis (*accA*,*B*,*C*,*D*, *fabD* and *fabH*) and activation of fatty acid synthase (*acp H*,*S*,*T*) but also in fatty acid catabolism (aerobic β-oxidation via *fadA*,*B*,*D*) and transport (*fadL*, a long-chain fatty acid outer membrane transporter) (S1 Table). Interestingly, fatty acid elongating genes (*fabG*, *fabZ*, *fabI*, *fabF*, *fabB* but not *fabA*) were also down-regulated. FabB is the first enzyme in the unsaturated fatty acid elongation pathway while FabF initiates elongation of the unsaturated fatty acid palmitoleate in cis-vaccenate. This general down-regulation of fatty acid metabolism could be directly linked to the general shortening of the acyl chain, shown in the presence of ionic silver. Moreover, although FabA and FabZ are isoform enzymes, only FabA can cause unsaturation in fatty acids. The relative level of FabA compared to that of FabB and FabZ controls the ratio of saturated to unsaturated fatty acids in *E*. *coli* [37, 38]. Fewer unsaturated bonds would be obtained at increasing concentrations of ionic silver if (i) FabA activity was lower than FabZ and/or if (ii) FabB activity was low. At the expression level, we found no such regulatory profiles, *fabA* was expressed at similar or higher levels than *fabZ* at 6.5 and 8.5 μM AgNO_3_ and *fabB* expression, despite its down-regulation, was not significantly lower than *fabA* (S2 Fig).

**Fig 6:**
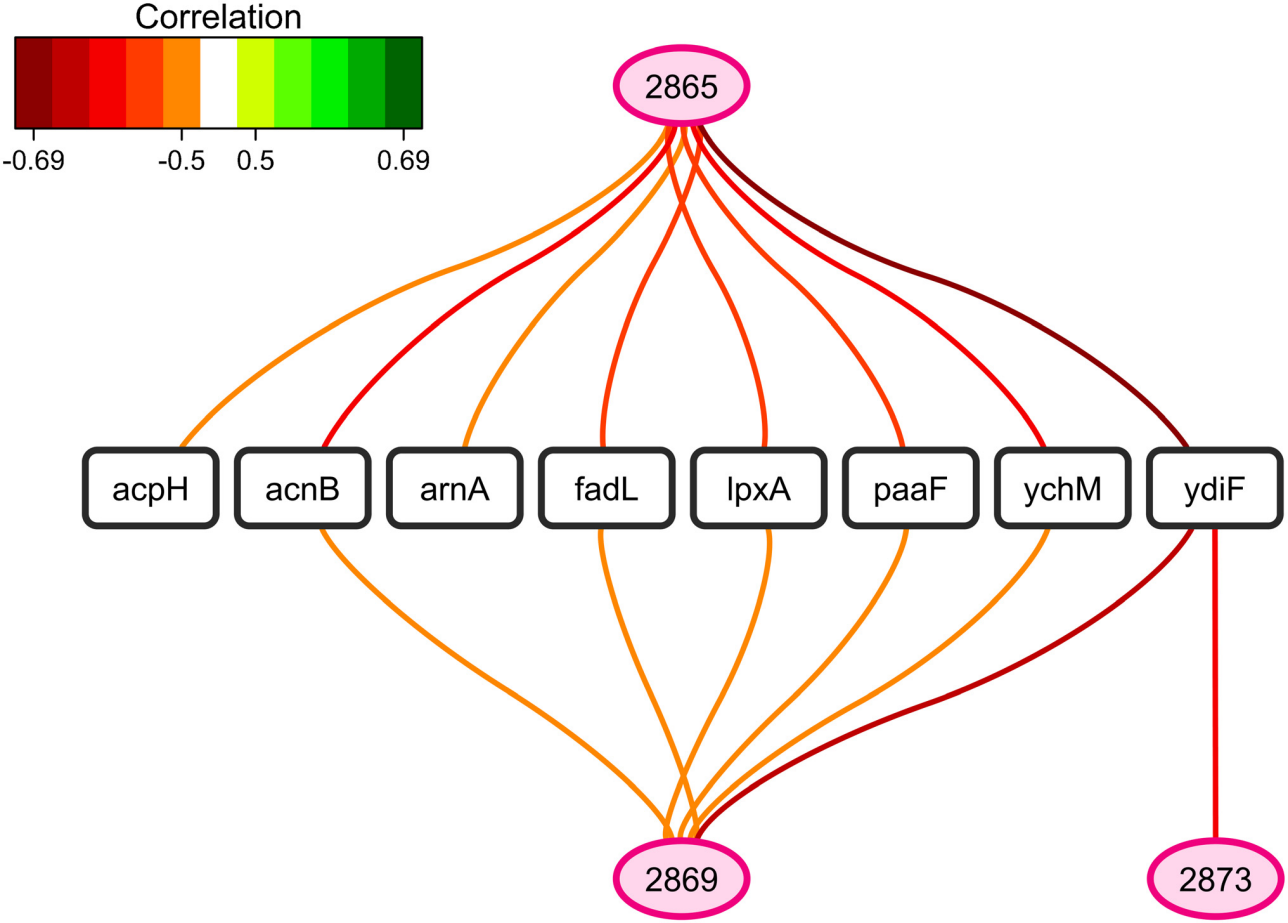
Relevance networks resulting from the sPLSR approach between the Y matrix composed of selected sFTIR data in the region of fatty acids (3,100–2,800 cm^-1^) and the X matrix corresponding to the expression of genes involved in fatty acid metabolism. The gene list was restricted to the 106 differentially-expressed genes annotated as involved in fatty acid and phospholipid metabolism and lipid transport (GO:0006631, GO:0006644 and GO:0006869). A threshold of 0.5 was used for relevant correlations. Green and red edges indicate positive and negative correlations respectively. Wavenumbers are represented as ellipses and genes as rectangles.

A search for correlations between selected sFTIR data and gene expression regulation revealed negative correlations between the fatty acid wavenumbers 2,873, 2,869 and/or 2,865 cm^-1^ and the expression of eight genes (Fig 6): *acpH* (of the *acpTSH* cluster involved in activation of fatty acid synthase), *acnB* (aconitase), *ydiF* (predicted acetyl-CoA transferase), *paaF* (of the phenylacetate metabolism) and *ychM* (predicted inner membrane transporter) and more interestingly, *fadL*, a long-chain fatty acid outer membrane transporter, *lpxA* and *arnA*, catalyzing biosynthesis and phosphate modification of lipid A, respectively. These results show that a higher peak at 2,873–2,865 cm^-1^ corresponding to shortening of an acyl chain was correlated with a lower concentration of fatty acid transporter at the outer membrane and (modified) lipid A.

Concerning protein folding, higher expression of chaperones *dnaJ* and *dnaK* was observed in Ag^+^-treated cells, whereas *groS* expression was less affected than the average (note that *groL* and *grpE* expression exhibited no specific regulatory pattern) (S1 Table). A marked decrease was observed in the expression of peptidylprolyl isomerase genes (*fklB*, *ppiB*, *surA* and *tig*), which function as protein folding chaperones for newly synthesised proteins. The bacterial response to low level of ionic silver was thus correlated with specific higher expression of chaperones involved in the rescue of misfolded proteins but not with those involved in folding newly formed proteins. Up-regulated expression of proteases (*lon*, *ftsH* and *htpX*) in the presence of ionic silver could be responsible for the degradation of unfolded/misfolded proteins. In the same way, expression of *clpA*,*P*,*X*, and *hslU*,*V* proteases did not follow the general trend of decreased expression. However, expression of *degP* involved in abnormal protein degradation in the periplasm nevertheless decreased.

**Fig 7:**
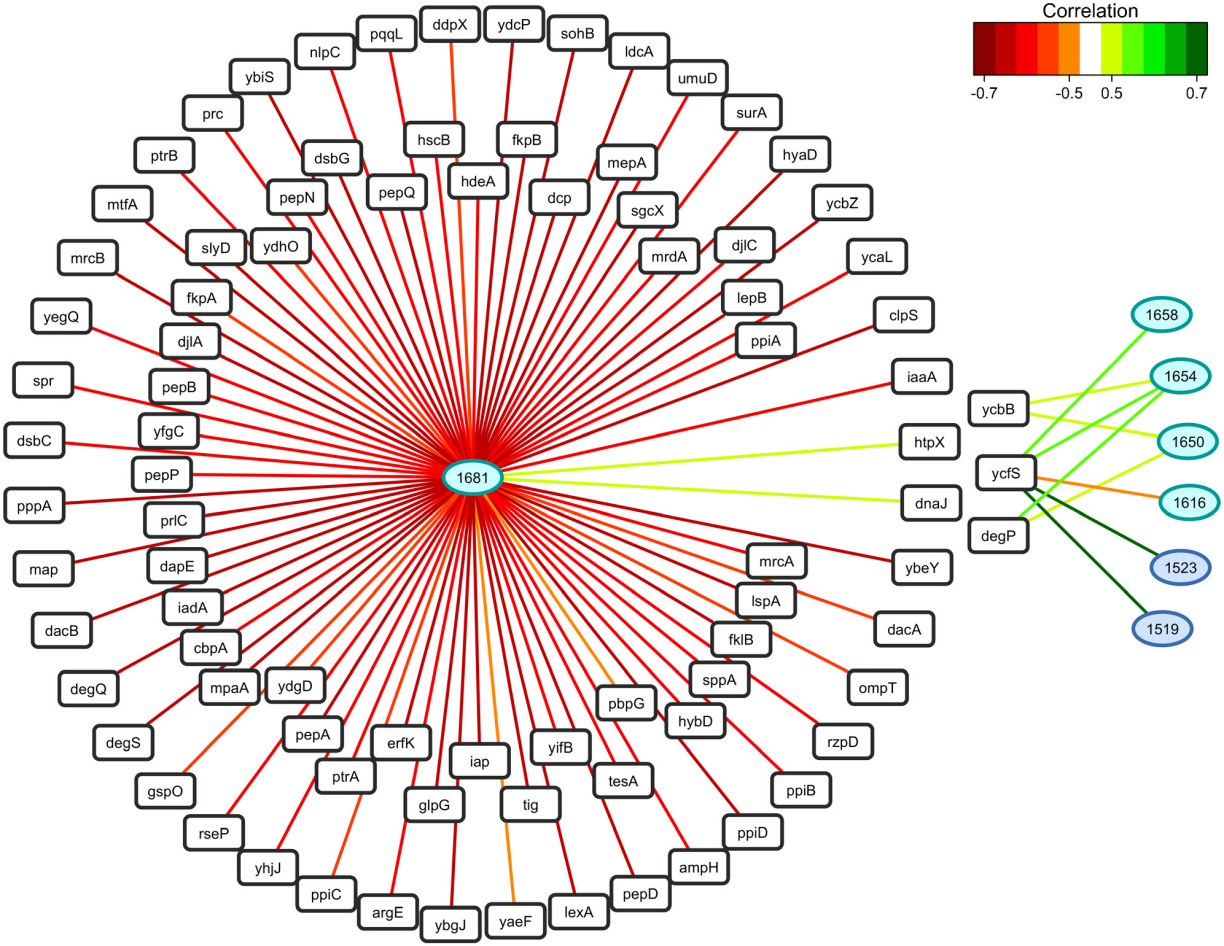
Relevance networks resulting from the sPLSR approach between the Y matrix composed of selected sFTIR data in the region of amide I and amide II (1,700–1,480 cm^-1^), and the X matrix corresponding to the expression of genes involved in protein folding. The gene list was restricted to the 104 differentially-expressed genes annotated as chaperone (GO:0051087), peptidase (GO:0008233), heat shock protein (GO:0031072), peptidyl-prolyl isomerase (GO:0003755) and protein disulfide isomerase (GO:0003756). A threshold of 0.5 was used for relevant correlations. Green and red edges indicate positive and negative correlations respectively. Wavenumbers are represented as ellipses and genes as rectangles.

As in the case of fatty acids, correlation analysis was performed to link variations in gene expression to protein structure-related sFTIR data (Fig 7). The up-regulated expression of protease *htpX* and chaperone *dnaJ* was identified as markers of the unordered random coil form of amide I (i.e., a positive correlation between expression regulation of *htpX* and *dnaJ* and the 1,681 cm^-1^ wavenumber). Concerning amide II, the β-sheet characteristic wavenumbers (1,519 and 1,523 cm^-1^) were not correlated with the expression of the previously mentioned protease and chaperone, but were strongly correlated with the L,D transpeptidase *ycfS*. The α-helix characteristic wavenumber 1,658 cm^-1^ was also positively correlated with *ycfS*, while wavenumbers 1,654 and 1,650 cm^-1^ were both positively correlated with the protease *degP*, and the L,D transpeptidase *ycbB*.

## Discussion

In this work, a multi-level characterization of *E*. *coli* response upon exposure to micromolar concentration of ionic silver was performed using an integrative biology approach to identify and connect different systemic effects at the transcriptional, physiological and biochemical levels.

First, it is important to note that the concentrations of AgNO_3_ used in this study were sub-lethal. Although cell growth was affected to different extents at 6.5 and 8.5 μM of AgNO_3_, after 3 hours of culture and contrary to a lethal concentration (e.g. a concentration of 100 μM of AgNO_3_ [15]), a proportion of the *E*. *coli* population (at least 10^7^ cells/mL/ODU at 6.5 and 8.5 μM of AgNO_3_) remained cultivable after removal of the silver. The effect of ionic silver on *E*. *coli* growth was shown to be, at least to some extent, linked to the accumulation of silver inside bacterial cells, as shown by NanoSIMS and elemental composition mapmaking. The overlap of silver with sulphur supported the hypothesis that Ag^+^ ions interact with intracellular proteins through the formation of silver-associated protein aggregates. The effect of ionic silver on proteins was further demonstrated (see below). To the best of our knowledge, this is the first time NanoSIMS has been used to probe silver intracellular uptake in prokaryotes, because previous studies were restricted to yeasts [22], daphnids [39, 40] and human cell lines [18, 40]. In fact, thanks to the excellent sensitivity of SIMS at nanoscale, it was possible to detect, localize, and quantify silver in small bacteria (through the Ag/CN ratio) at very low concentrations, i.e. when 5.0 μM AgNO_3_ was added to the medium. Notably, it was shown that a tiny increase in intracellular silver content between cells treated with 5.0 or 6.5 μM of AgNO_3_ could affect cell growth. This may be due to the phenomenon of silver "bioaccumulation", which explains the efficacy of the Ag^+^ ion even at low micromolar concentrations [2]. As reported previously [41], the amount of intracellular ionic silver was a better marker of the toxicity of silver nanoparticles than the quantity of Ag^+^ ions dissolved from these nanoparticles in the bacterial environment. This corroborates the interest of NanoSIMS for detecting intracellular bioavailable silver. For bacterial cells treated with 8.5 μM AgNO_3_, the Ag/CN ratio was lower, probably indicating intracellular silver release due to damaged and more “leaky” *E*. *coli* cell walls. This type of morphological alterations have already been described [1] in *E*. *coli* cells exposed to 10 μg/mL AgNO_3_ (i.e., 92.7 μM) as well as in our earlier study [15]. It can also be hypothesised that an active system of transport was set up by cells to excrete silver [12].

A search for correlations between sFTIR data and intracellular silver content confirmed our previous conclusions obtained with the extracellular AgNO_3_ concentration, in particular silver-mediated shortening of acyl chains in fatty acids of *E*. *coli* after exposure to sub-lethal concentrations of ionic silver (i.e., < 10 μM) [15]. However, in the present study, our insight into intracellular silver composition and the use of a multivariate statistical data analysis for identification of covariance (sPLSR) allowed more subtle conditions to be correctly probed and distinguished, thereby enhancing our initial approach, which only took the extracellular concentration of AgNO_3_ into account and was based on the combination of principal component analysis (PCA) and partial least squares (PLS) regression. In particular, variations in the conformation of unsaturated fatty acids were highlighted. In fact, in the presence of low concentrations of intracellular silver (i.e., sub-lethal AgNO_3_ concentrations) and in agreement with the results of our earlier study [15], a higher “cis” conformation content was observed, indicating a slight increase in bacterial membrane fluidity. It can be hypothesised that these changes in membrane fluidity facilitate silver excretion and correspond to an active response by bacterial cells. Many studies focused on the membrane unsaturated fatty acids of bacterial cells subjected to various adverse environments have reported “cis-to-trans” isomerisation (for example, [42] in *E*. *coli*; [43], [44] and [45] *Pseudomonas* or *Vibrio*), further illustrated by our group in *E*. *coli* at a lethal concentration of silver (100 μM AgNO_3_) [15]. However, an increase in “cis” conformation was also reported by [46], in a study of the effects of sub-lethal concentrations of eugenol on the fatty acid profile of *E*. *coli* O157:H7 ATCC 43888 membranes. In a previous study [47], the same authors also observed this type of modification by evaluating the stress induced by a sub-lethal mix of ethanol and hexanal on the membrane fatty acid composition of *E*. *coli* 555. Alterations of the protein secondary structure (i.e., loss of α-helix structures) were clearly demonstrated in the present study, while not detected at similar AgNO_3_ concentrations in our earlier work [15]. Lastly, new findings, such as the disruption of lipid esters, were also obtained thanks to our optimised and more sensitive method.

The transcriptomic response was weak at 5.0 μM but massive and strong at 6.5 μM and 8.5 μM AgNO_3_ with a majority of down-regulated genes mainly involved in growth-related functions. Down-regulated expression of transporter genes, transcriptional regulators and ribosomal proteins was positively correlated with the decrease in the growth rate and in the number of cultivable cells. Although in our study, the global transcriptomic response generally differed from the copper-mediated one, we showed that the AgNO_3_ concentration and Ag/CN ratio were positively correlated with up-regulation of genes specifically involved in the response to copper stress. This confirms that mechanisms to cope with silver and copper share a primary implication of the efflux transport *copA* in the resistance process and oxidation via the cuproux oxidase CueO [16]. But we also showed that the ionic silver concentration was positively correlated with up-regulated markers of the general stress response, cation transport and methionine biosynthesis. The general response to stress contributes to the response to silver as shown in *E*. *coli* cells exposed to Zn [48], ethanol and sodium hydroxide [42]. In addition, our integrative approach revealed that within the general stress response, up-regulated expression of chaperones and proteases in the presence of silver ions was correlated with the increase in disordered secondary structure of proteins.

Only global transcriptomic responses associated with growth arrest conditions were strongly correlated with one another, indicating that the global variation in gene expression reflects a growth rate effect rather than a direct effect of silver. Therefore, at concentrations of 6.5 μM and 8.5 μM of AgNO_3_ corresponding to non-growing cells, the observed genetic response probably corresponded to the overlap between the direct specific transcriptional responses to coping with ionic silver stress and the indirect general transcriptional response linked to changes in the growth rate. In the future, it would thus be interesting to conduct a study of *E*. *coli* response to ionic silver-mediated stress in chemostat culture at imposed growth rates in order to remove the growth rate effect and specifically focus on the silver-mediated effect [49].

Of note, under our experimental conditions, we did not find any clear specific up-regulated expression of the DNA repair processes (excision repair, nucleases, recombination and error-prone DNA polymerase) or of anaerobic respiration after exposure to silver ions, as previously reported for silver nanoparticles [11, 17]. On the contrary, we found strong down-regulation of genes involved in anaerobic respiration (nitrate/nitrite reductase, fumarate reductase enriched in clusters 3 and 4, Fig 4). Consequently, the antibacterial effect of ionic silver appears to differ, at least to some extent, from the effect exerted by nano-sized silver, as recently reported [41]. Furthermore, the down-regulated expression of many oxidase-reductase genes was shown to be correlated with the presence of silver ions. This finding, added to the correlation found between the AgNO_3_ concentration and up-regulation of methionine biosynthesis, strongly suggests the occurrence of oxidative stress in bacterial cells exposed to ionic silver [41, 50, 51]. Taken together, these results show that the response to ionic silver stress at the transcriptomic level corresponds to a particular combination of components involved in a range of different responses (growth rate, metal ion stress, general stress and oxidative stress).

The effect of ionic silver, i.e. reducing the amount of unsaturated fatty acids [15], was shown here to be not related to the transcriptomic regulation of the three key enzymes FabA, FabB and FabZ. However, we cannot exclude the possibility that ionic silver plays a role in regulating the synthesis (at the post-transcriptional level) and the activity (at the post-translational level) of these enzymes. Furthermore, the silver-induced shortening of the acyl chain length was shown to be correlated with the down-regulated expression of genes involved in the unsaturated fatty acid elongation pathway, fatty acid synthesis and transport, and also catalysing biosynthesis and phosphate modification of lipid A. This result clearly shows that cell membrane is a key target of ionic silver. In addition, in an earlier study by our group, the cell ultrastructure of *E*. *coli* was shown to be damaged under Ag^+^-mediated stress, with possible detachment of the outer membrane at lethal and sub-lethal concentrations of ionic silver [15]. Using correlation analysis of changes in gene expression and protein structure-related sFTIR data, we are now able to provide a plausible explanation for these morphological changes. Indeed, the conformational shift (from α-helix to β-sheet) of amides I and II in the presence of ionic silver was correlated with gene expression regulation of L,D transpeptidases YcfS and YcbB. Interestingly, these L,D transpeptidases act on the structural integrity of the cell wall. In particular, YcfS contributes to the covalent attachment of peptidoglycan to the outer membrane whereas YcbB is involved in the synthesis of peptidoglycan cross-links [52]. *ycfS* expression was up-regulated in our experiments, while *ycbB* expression was less affected than the average of all other genes. Therefore, the specific regulation of the expression of YcfS and YcbB in the presence of ionic silver could be at play in the morphological changes observed. Therefore, a molecular approach aimed at modulating the expression of YcfS and YcbB can be recommended to control the effects of silver on *E*. *coli* when ionic silver is used in antibacterial coatings, for instance.

In conclusion, the multi-level characterization of *E*. *coli* exposed to Ag^+^-mediated stress was achieved using an integrative biology approach, based on the combination of physiological, biochemical and transcriptomic data sets. From an analytical point of view, the elemental composition mapmaking provided by NanoSIMS was used for the first time to unambiguously detect and localize intracellular silver within individual bacterial cells in interaction with cell components like proteins, due to NanoSIMS high sensitivity and sub-micrometer lateral resolution. The semi-quantitative data obtained with this powerful tool combined with the evaluation of biochemical composition of the cell at the single-cell scale thanks to sFTIR microspectroscopy was extremely useful in further explaining the physiological results. Finally, it was possible to define a genetic pattern of the ionic-silver induced response specifically dedicated to coping with intracellular silver but also associated with changes in growth and survival, cell membrane and protein composition and structure. As Gram-positive bacteria are generally said to be less sensitive to ionic silver than Gram-negative bacteria, notably due to differences in the structure of the cell wall, it would be interesting to use the same approach to explore the biological response of Gram-positive bacteria under silver stress.

## Supporting Information

S1 Fig (A) **FTIR raw spectra** (means of 20 spectra) in the 4,000–800 cm^-1^ region recorded at a single-cell scale in *E*. *coli* grown without (control) or in the presence of ionic silver at sub-lethal concentrations. The regions of interest are identified in the figure. (B) Focus on the fatty acid region (3,100–2,800 cm^-1^) with labels on the peaks attributed to the symmetric C-H stretching of—CH_3_ and >CH_2_ groups and to the up- and downshift of the asymmetric C-H stretching of >CH_2_ bands. (C) Focus on the region including the bands attributed to C = O of esters, amides I and II and PO_2_^-^ of nucleic acids and C-O stretching modes (1,800–1,200 cm^-1^). The bands characteristic of C = O in lipid esters, amide I of peptide bonds and NH_2_ of adenine are labeled.
(TIF)

S2 Fig Expression of *fabA*, *fabZ* and *fabB* in response to ionic-silver mediated stress. Intensity (in log_2_) reflects the level of gene expression in each growth condition, i.e. with 0, 5.0, 6.5 and 8.5 μM of AgNO_3_ in the medium.
(TIF)

S1 Table Fold changes in expression and p-values between different concentrations of AgNO_3_ and the associated cluster number for all the genes. (XLSX)
