# Supplementary figures and images for: Escherichia coli under Ionic Silver Stress: An Integrative Approach to Explore Transcriptional, Physiological and Biochemical Responses
Source: PLoS One. 2015 Dec 22;10(12):e0145748. doi: 10.1371/journal.pone.0145748 (PMC4699211; doi:10.1371/journal.pone.0145748)

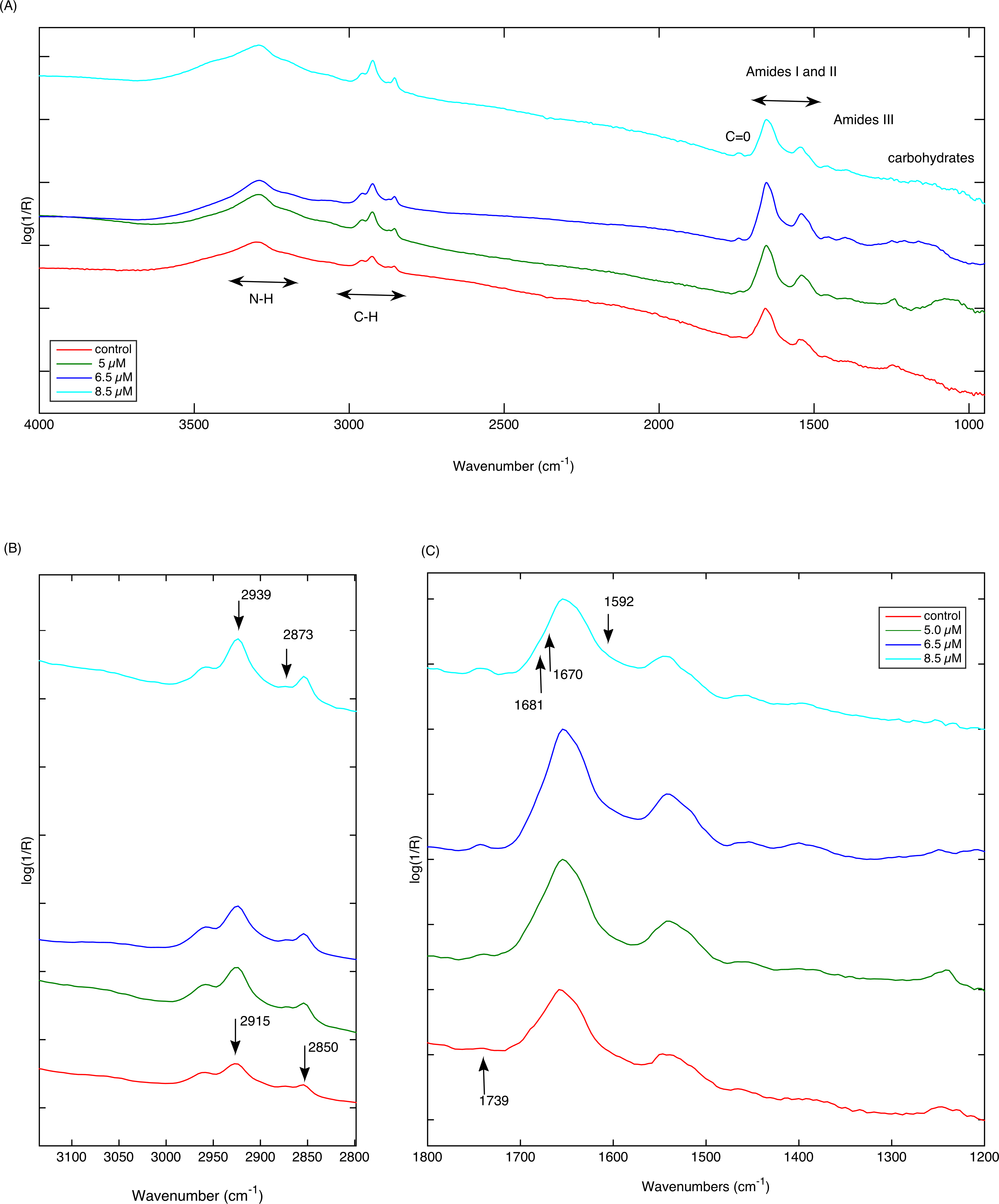

Supplement: S1 Fig — (A) FTIR raw spectra (means of 20 spectra) in the 4,000–800 cm-1 region recorded at a single-cell scale in E. coli grown without (control) or in the presence of ionic silver at sub-lethal concentrations. The regions of interest are identified in the figure. (B) Focus on the fatty acid region (3,100–2,800 cm-1) with labels on the peaks attributed to the symmetric C-H stretching of—CH3 and >CH2 groups and to the up- and downshift of the asymmetric C-H stretching of >CH2 bands. (C) Focus on the region including the bands attributed to C = O of esters, amides I and II and PO2 - of nucleic acids and C-O stretching modes (1,800–1,200 cm-1). The bands characteristic of C = O in lipid esters, amide I of peptide bonds and NH2 of adenine are labeled. (TIF) [file pone.0145748.s001.tif]

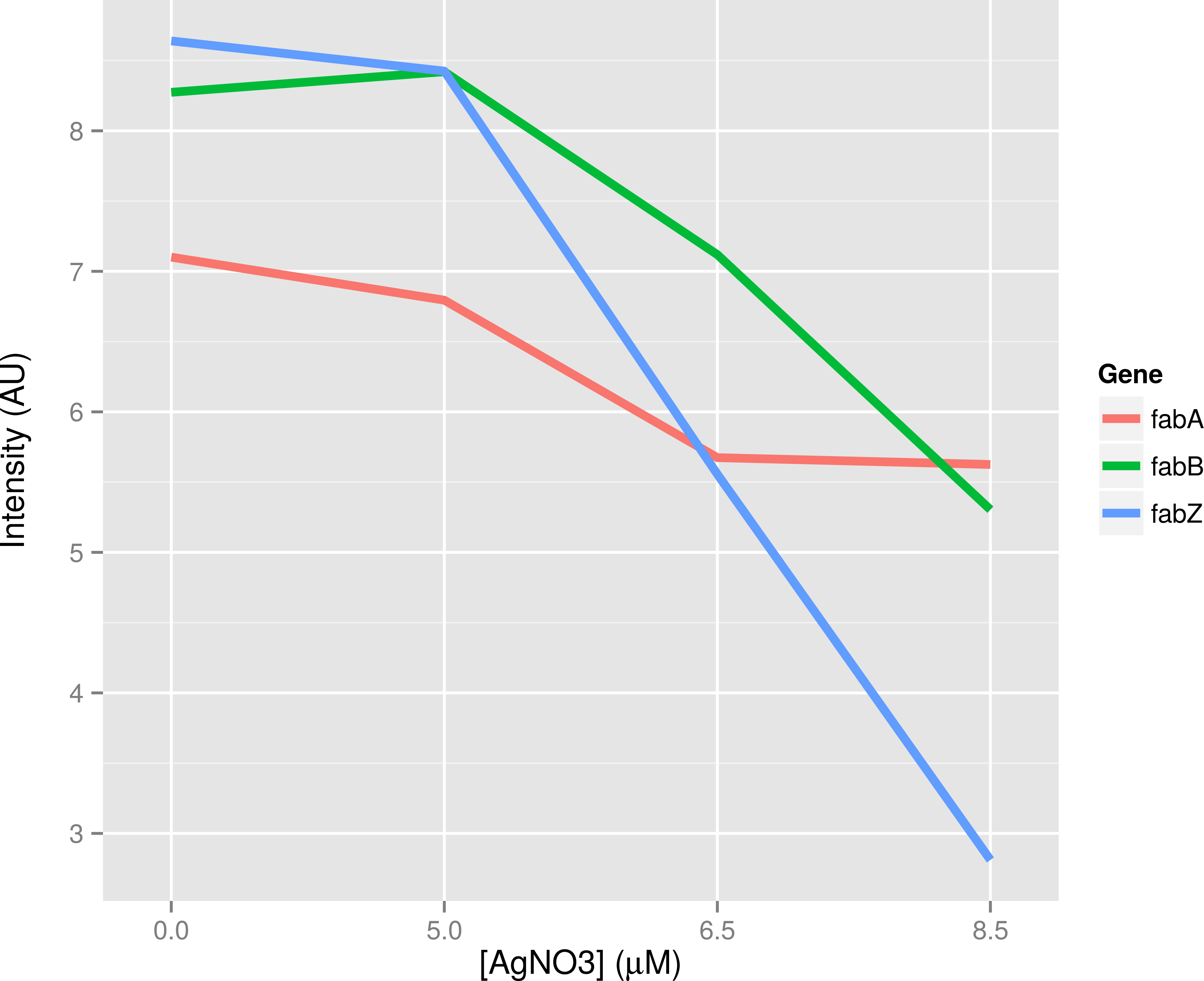

Supplement: S2 Fig — Intensity (in log2) reflects the level of gene expression in each growth condition, i.e. with 0, 5.0, 6.5 and 8.5 μM of AgNO3 in the medium. (TIF) [file pone.0145748.s002.tif]
